# Do We Know What We Enjoy? Accuracy of Forecasted Eating Happiness

**DOI:** 10.3389/fpsyg.2020.01187

**Published:** 2020-06-17

**Authors:** Karoline Villinger, Deborah R. Wahl, Laura M. König, Katrin Ziesemer, Simon Butscher, Jens Müller, Harald Reiterer, Harald T. Schupp, Britta Renner

**Affiliations:** ^1^ Department of Psychology, University of Konstanz, Konstanz, Germany; ^2^ Department of Computer and Information Science, University of Konstanz, Konstanz, Germany

**Keywords:** affective forecasting, forecasting accuracy, individual differences, impact bias, eating happiness

## Abstract

Forecasting how we will react in the future is important in every area of our lives. However, people often demonstrate an “impact bias” which leads them to inaccurately forecast their affective reactions to distinct and outstanding future events. The present study examined forecasting accuracy for a day-to-day repetitive experience for which people have a wealth of past experiences (eating happiness), along with dispositional expectations toward eating (“foodiness”). Seventy-three participants (67.12% women, *M*_age_ = 41.85 years) used a smartphone-based ecological momentary assessment to assess their food intake and eating happiness over 14 days. Eating happiness experienced in-the-moment showed considerable inter-and intra-individual variation, *ICC* = 0.47. Comparing forecasted and in-the-moment eating happiness revealed a significant discrepancy whose magnitude was affected by dispositional expectations and the variability of the experience. The results demonstrate that biased forecasts are a general phenomenon prevalent both in outstanding and well-known experiences, while also emphasizing the importance of inter-individual differences for a detailed understanding of affective forecasting.

## Introduction

How we think we will feel in response to future events or behaviors is often crucial in the decisions we make in all areas of our lives. Predictions about our future emotions, termed “affective forecasting” by Wilson and Gilbert (2003, 2013), can impact important life-decisions such as starting a new job or getting married, and also more mundane everyday decisions such as our choice of food. Forecasting affective reactions to future events or behaviors is therefore of fundamental importance in the process of decision making (Mellers et al., 1999; Mellers, 2000; Mellers and McGraw, 2001; Loewenstein and Lerner, 2003; Kermer et al., 2006; Gilbert and Wilson, 2009; Hoerger et al., 2012a).

People are generally quite good at forecasting the overall valence or nature of their emotional reactions (Robinson and Clore, 2001; Wilson et al., 2002 as cited in Wilson and Gilbert, 2003). They can usually foretell whether eating sushi will be a positive or negative experience, and if they will find it pleasurable or disgusting. However, when forecasting the relative impact of future emotional events, people tend to overestimate how intensive their feelings will be and how long they will last (Wilson and Gilbert, 2003).

The tendency to overestimate the initial impact in terms of the felt intensity and/or duration of an emotional event has been termed “impact bias” (see Wilson and Gilbert, 2003 for an overview). For instance, people tend to overestimate the impact of negative events, such as how negatively and for how long they will be emotionally affected by the breakup of a relationship or a setback in their career. The duration and intensity of experienced emotional reactions toward positive events such as one’s favorite football team winning or going on vacation are also often overestimated (Gilbert et al., 1998; Wilson et al., 2000; Wirtz et al., 2003). The impact bias has been shown within various populations and in a wide range of domains ranging from personal insults, fear, pain, the end of a romantic relationship, the results of clinical tests, and academic performance to vacation experiences, sport events, and election outcomes (Rachman, 1994; Mitchell et al., 1997; Gilbert et al., 1998; Sieff et al., 1999; Buehler and McFarland, 2001; Mellers and McGraw, 2001; Woodzicka and LaFrance, 2001; Wilson and Gilbert, 2003; Wirtz et al., 2003; Hoerger et al., 2009).

Most studies examining affective forecasting used comparably distinct and outstanding events such as a specific football match or the spring break vacation and involved relatively confined and short-term laboratory paradigms such as eating a single provided meal (Buehler and McFarland, 2001; Dunn et al., 2003; Wilson et al., 2003; Wilson and Gilbert, 2005; Robinson et al., 2013; Robinson, 2014). Few studies have used a long-term, real-life ecological setting to examine the relationship between forecasted and experienced emotions (Klaaren et al., 1994 (Study 1); Mitchell et al., 1997; Wirtz et al., 2003). For example, Wirtz et al. (2003) examined the relationship in a sample of college students regarding their spring break experience and found that forecasts of how they expected to experience the vacation were both more positive and more negative than the actual experiences in-the-moment. These biased views about how much they would enjoy or dislike their holidays replicates the impact bias in a long-term, real-life ecological setting.

Wirtz et al. (2003) argued that people might hold general beliefs or assumptions about the event as affectively intense which disregards hedonically neutral moments, resulting in overestimations of the intensity of the actual experience. One could similarly argue that distinct and outstanding events such as spring break vacations elicit forecasting errors because people lack prior experiences and need to rely on general beliefs and stereotypes to make affective forecasts (Wilson and Gilbert, 2003). In contrast, familiar day-to-day events such as food choices and eating, or social interactions, offer a wealth of concrete, individual experiences ranging from very positive to very negative, which may lead to greater forecasting accuracy.

As with distinct and outstanding events, affective experiences of familiar day-to-day events are likely to differ between individuals. Importantly, since the same individual is constantly experiencing familiar day-to-day events, inter-individual differences in the within-person variability of the experienced affective responses are also likely to emerge. The variability of experiences might impact forecasting accuracy, since it is theoretically more difficult to forecast fluctuating experiences across time and/or events than relatively stable and consistent ones. However, so far, the research of affective forecasting has not considered the impact of the variability of experiences as a central aspect. Accurate affective forecasting of long-term, real-life ecological settings might therefore also depend on inter-individual differences in the intra-individual variability of affective reactions toward events (see also Neubauer et al., 2019).

Furthermore, besides inter-individual differences in the intra-individual variability of affective reactions (see also Augustine and Larsen, 2012; Brans et al., 2013), research suggests that dispositional differences and personality play an important role in affective forecasting as they might affect both forecasts and the experience itself (e.g., Hoerger et al., 2012a; Zelenski et al., 2013). For example, people hold general beliefs or assumptions about themselves and their future experiences, as well as events. Specifically, numerous studies have shown that people differ in their viewpoint about what the future will hold for them, described by Carver and Scheier (2014) as their “mental orientation to experiences.” People who generally endorse a more positive outlook on their future experiences as a facet of their personality might consequently make more positive forecasts for specific, circumscribed events. Moreover, dispositional expectations might not only affect forecasts of the intensity of affective experiences, but might also change the actual affective reaction to the event itself (expectation effect, Wilson et al., 1989; Wilson and Gilbert, 2003). Accordingly, a greater covariation between forecasted and experienced affect might emerge when individual differences in dispositional expectations are also taken into account.

The aim of the present study was to investigate affective forecasting accuracy in familiar day-to-day experiences in a long-term, real-life ecological setting. Specifically, we assessed eating-induced affect experienced in-the-moment using a 2-week event-based ecological momentary assessment (EMA; Shiffman et al., 2008; Trull and Ebner-Priemer, 2013, 2014; Conner and Mehl, 2015; Wahl et al., 2017a) to capture the variability and diversity of experiences. Forecasted eating happiness assessed before the EMA period was compared with aggregated eating happiness experienced in-the-moment to measure forecasting accuracy. In the area of eating and experienced eating happiness, inter-individual differences in the “mental orientation to experiences” and related dispositional expectations might manifest in how much people enjoy their meals and eating in general, which can be conceptualized as their general level of “foodiness.”

In the present research, we tested three hypotheses derived from the literature presented. First, consistent with the impact bias, forecasted eating happiness should be more positive than eating happiness assessed in-the-moment (“impact bias”). Second, dispositional expectations (“foodiness”) are expected to moderate eating happiness and the observed forecasting accuracy. Third, forecasting accuracy is expected to be related to the intra-individual variability of affective reactions toward eating occasions. Specifically, a greater variability in affective reactions toward eating occasions should result in a lower forecasting accuracy.

## Materials and Methods

The present study was part of the SMARTACT research project, funded by the Federal Ministry of Education and Research (BMBF, Grant 01EL1820A)^1^. The study was pre-registered at the DRKS (ID DRKS00010279) and conformed to the guidelines of the German Psychological Society (Deutsche Gesellschaft für Psychologie) and the Declaration of Helsinki. The study protocol was approved by the University of Konstanz’s Institutional Review Board, and adhered to ethical guidelines and regulations. All participants gave written, informed consent prior to participation.

### Participants

A total of 96 individuals were recruited for the study. Inclusion criteria were being of age (18 years or older), willingness to use a smartphone, fluency in German language, and a previous participation at the Konstanz Life Study in 2016. Of the initial 96 participants, 16 withdrew their participation due to illness or other constraints. One participant had to be excluded due to missing data on forecasted eating happiness. Furthermore, analysis of frequency of reported eating occasions revealed a bimodal distribution. Consistent with previous findings on eating occasions (Ziesemer et al., 2020), the majority of participants recorded more than three eating occasions per day (*M* = 3.65 and *SD* = 1.38). However, six participants reported on average less than one eating occasion per day over the study period and therefore were excluded from data analysis, as frequency of eating occasions was considered too low for meaningful data analysis. A final sample of 73 participants was included in the analysis (67.12% women) with a mean age of 41.85 (*SD* = 15.21 and 20–78 years) and an average BMI of 24.88 (*SD* = 3.93 and 17.92–38.01 kg/m^2^). Overall, the sample was healthy with participants rating their general health status as good (*M* = 3.77 and *SD* = 0.79), on a scale ranging from very bad (1) to very good (5) and their eating behavior as rather healthy (*M* = 2.81 and *SD* = 0.70) and balanced (*M* = 2.97, *SD* = 0.833), both on a scale ranging from very healthy/balanced (1) to very unhealthy/unbalanced (6). As compensation, participants received a detailed written feedback about their eating behavior and eating profile characteristics.

### Procedure

The participants were recruited during August and October 2016 from the fourth wave of the Konstanz Life Study, a longitudinal cohort study (Renner et al., 2012), and stratified according to age and gender. They were invited to the university in small groups of 2–12 people for introduction sessions, during which they completed a questionnaire assessing their dispositional and forecasted eating happiness, and their anthropometric measures were recorded. They were also familiarized with the smartphone and the preinstalled “SMARTFOOD” application (app) and given a booklet which explained how to use the smartphone and the app to record their food intake.

Each participant was provided with a study smartphone (ASUS Padfone Infinity, Android 5.0.2 or Samsung Galaxy J5, Android 6.0.1), and used the mobile application “SMARTFOOD” developed as part of the research project SMARTACT^1^ (for more details, see Butscher et al., 2016; Villinger et al., 2017; Wahl et al., 2017b), to record eating occasions and eating happiness in-the-moment of consumption. The participants were instructed to record all eating occasions, including meals and snacks, for 14 consecutive days, and specifically to assess the meal types (breakfast, lunch, teatime, dinner, and snack), take pictures of each eating occasions (including initial portion, additional courses, and leftovers), and select the main components of the meal using a search function. They were further asked to rate the eating happiness they experienced in-the-moment of eating immediately after their meal was finished. Ratings were final and participants were not able to change their ratings after they submitted the responses. To ensure that participants remembered to access the app after they finished eating, a reminder was sent automatically after 20min if assessment had not been finalized within the app.

### Measures

#### Eating Happiness Experienced In-the-Moment

During each eating occasion, participants rated (1) how much they enjoyed their meal, (2) how pleased they were with their meal, and (3) how tasty their meal was on a 100-point visual slider raging from “not at all” (0) to “a lot” (100) (see also Wahl et al., 2017a). Responses to the three items were highly interrelated (mean Cronbach’s *α* = 0.91), and therefore the items were averaged to create a composite score.

#### Forecasted Eating Happiness

Before the EMA period, participants forecasted their eating happiness by responding to the header “In the following weeks, during the study, I expect… (1) to enjoy my meals, (2) to be pleased with my meals, and (3) that my meals will be tasty.” Responses were given on a 100-point visual slider ranging from “not at all” (0) to “a lot” (100). As responses were highly interrelated (Cronbach’s *α* = 0.92) an average forecasted eating happiness composite score was calculated for the analysis.

#### Affective Forecasting Accuracy

**Figure 1:**
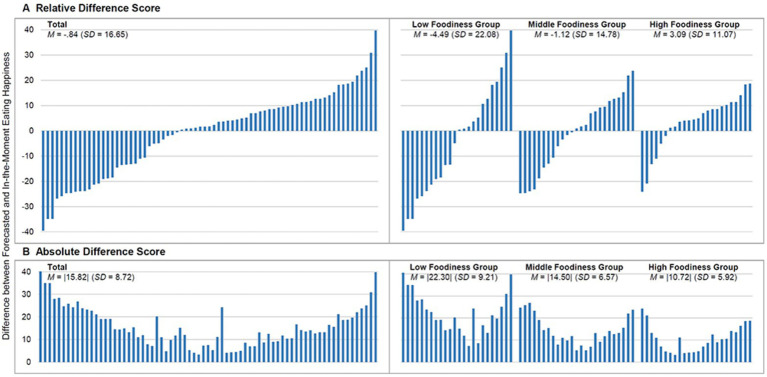
Difference score between forecasted and in-the-moment eating happiness for each participant and by foodiness group. Higher values indicate a greater difference score (= lower forecasting accuracy). **(A)** relative difference score. **(B)** absolute difference score.

Affective forecasting accuracy was calculated by assessing the difference between forecasted and eating happiness experienced in-the-moment (*M*_forecasted_−*M*_in-the-moment_) for each eating occasion separately and then aggregating across all eating occasions per participant, referred to as relative difference score. The analyzes at individual and group levels revealed a mutual rescind of the individual deviations between forecasted and in-the-moment eating happiness (see also Figure 1). Therefore, in a second step, the absolute value of the difference between forecasted and eating happiness experienced in-the-moment (|*M*_forecasted_−*M*_in-the-moment_|) was calculated for each eating occasion and aggregated per participant as described above, referred to as absolute difference score (c.f., Dunn et al., 2007; Hoerger et al., 2012a, 2016).

#### Dispositional Eating Happiness (“Foodiness”)

Dispositional eating happiness was assessed before the EMA period by the following statements “In general… (1) I enjoy my meals, (2) I am pleased with my meals, and (3) my meals are tasty.” Responses were made on a 100-point visual slider anchored at “not at all” (0) to “a lot” (100). As responses were highly interrelated (Cronbach’s *α* = 0.84) an average dispositional eating happiness composite score was calculated for the analysis and categorized with a tertian split as “low” (score < 67; *M* = 56.01 and *SD* = 6.14), “middle” (score 67–82, *M* = 73.67 and *SD* = 3.78), and “high” (score > 82; *M* = 90.19 and *SD* = 4.62) dispositional eating happiness (“foodiness”). Dispositional eating happiness correlated with forecasted eating happiness with *r* = 0.56 and *p* < 0.001.

### Analytical Procedure

Based on empirical suggestions about effect sizes using mobile health apps (Kerr et al., 2012) and an expected drop-out rate around 35% we target a sample size of *N* = 100. To test forecasting accuracy, one sample *t*-tests against zero were conducted, with zero representing no divergence between forecasted and in-the-moment eating happiness. Group differences in eating happiness and forecasting accuracy were examined via separate ANOVAs and subsequent *post-hoc* analyzes (Bonferroni) to assess the impact of dispositional expectations. Variability of in-the-moment eating happiness was analyzed with intra-class correlations (ICC). In addition, experienced variability between eating occasions and assessment days was calculated per participant and subsequently used to assess the impact of experienced variability on forecasting accuracy via linear regressions. For this purpose, variance between eating occasions was calculated per participant taking experienced eating happiness of all eating occasions over the 14-day period into account. Furthermore, to assess the variability between days, we calculated the average of eating happiness per day and determined the variance between days. Regressions were controlled for group membership and conducted separately for variability between eating occasions and assessment days. Effect sizes were calculated using Cohen’s *d*, *η* ^2^, and standardized *β* for the respective analyzes (Cohen, 1988). Analyzes were conducted with IBM SPSS Statistics (version 24 for Windows), and graphically visualized using Tableau (version 10.1).

## Results

### Eating Happiness Experienced In-the-Moment

A total of 2,898 eating occasions were reported during the 2-week assessment period. On average, participants recorded 3.65 eating occasions per day (*SD* = 1.38). As Table 1 shows, eating happiness experienced in-the-moment associated with these eating occasions ranged from a low of 2.67 to a high of 100.00, with an average of *M* = 81.09 (*SD* = 16.67). In-the-moment eating happiness differed between foodiness groups, *F*(2,70) = 8.89, *p* < 0.001, and *η* ^2^ = 0.20. Participants with a generally more positive outlook about their future eating experiences (“high foodiness”) showed a significant higher in-the-moment eating happiness than those with a more negative outlook (“low foodiness”), *p* < 0.001.

**Table 1:** Eating happiness experienced in-the-moment for the total sample and by foodiness group (dispositional eating happiness).

Participants	Eating occasions *N* (%)	*M*	*SD*	Range	*ICC*
Total (*N* = 73)	2,898 (100%)	81.09	16.67	2.67–100	0.47
Low foodiness group (*n* = 24)	872 (30.10%)	73.76	18.62	2.67–100	0.40
Middle foodiness group (*n* = 25)	966 (33.30%)	80.98	14.32	15.33–100	0.43
High foodiness group (*n* = 24)	1,060 (36.60%)	87.23	14.37	28.33–100	0.42

*ICC*, intraclass correlation. Descriptive data and ICC analyses are based on a long-format of the data with *df* = 2,897.

### Comparing Forecasted and In-the-Moment Eating Happiness

#### Analysis of Relative Difference Scores

For all participants, forecasted eating happiness did not deviate significantly from in-the-moment eating happiness [*t*(72) = −0.43, *p* = 0.666, and *d* = 0.06]. Comparisons within foodiness groups also revealed no significant discrepancies between forecasted and in-the-moment eating happiness (see Table 2).

**Table 2:** Type of eating happiness and forecasting accuracy for the total sample and by foodiness group (dispositional eating happiness).

Participants	Total (*N* = 73)	Low foodiness group (*n* = 24)	Middle foodiness group (*n* = 25)	High foodiness group (*n* = 24)
Eating happiness				
*M_disp_* (*SD*)	73.30 (14.78)	56.01 (6.14)	73.67 (3.78)	90.19 (4.62)
*M_forec_* (*SD*)	79.90 (15.90)	69.91 (17.22)	79.48 (12.87)	90.33 (10.19)
*M_mom_* (*SD*)	80.75 (11.65)	74.40 (12.07)	80.60 (9.77)	87.24 (9.68)
Forecasting accuracy				
Relative difference score				
*M_forec_*−*M_mom_* (*SD*)	−0.84 (16.65)	−4.49 (22.08)	−1.12 (14.78)	3.09 (11.07)
*t* (*p*)	−0.43 (0.666)	−1.00 (0.326)	−0.38 (0.708)	1.37 (0.185)
*d*	0.06	0.30	0.10	0.31
Absolute difference score				
|*M_forec_*−*M_mom_*| (*SD*)	|15.82| (8.72)	|22.30| (9.21)	|14.50| (6.58)	|10.72| (5.92)
*t* (*p*)	15.51 (<0.001)	11.86 (<0.001)	11.03 (<0.001)	8.87 (<0.001)
*d*	1.81	2.42	2.20	1.81

Person-mean of eating happiness experienced in-the-moment was used for analysis. *M_disp_*,mean of dispositional eating happiness; *M_forec_*, mean of forecasted eating happiness; *M_mom_*, mean of eating happiness experienced in-the-moment; forecasting accuracy: higher values indicate a greater difference score (= lower forecasting accuracy).

As Figure 1A displays, there were pronounced inter-individual differences between forecasted and in-the-moment eating happiness. Overall, 30 participants forecasted a more positive and 43 a less positive eating happiness than they actually experienced in-the-moment of eating. A similar picture emerged within each foodiness group (see Figure 1A, right side). A comparable number of participants in the low and middle foodiness groups made higher and lower forecasts of their eating happiness than they actually experienced in-the-moment, resulting in an inverse structure across participants within the groups. In contrast, 18 participants from the high foodiness group forecasted a higher and only six a lower eating happiness than they experienced in-the-moment.

#### Analysis of Absolute Difference Scores

Due to the mixed pattern of overestimations and underestimations, the size of the difference between forecasted and in-the-moment eating happiness was analyzed. The absolute difference score indicates that forecasted eating happiness deviated significantly from in-the-moment eating happiness, *M* = |15.82|, *SD* = 8.72, *t*(72) = 15.51, *p* < 0.001, and *d* = 1.81 (Table 2). Importantly, there was a considerable variation in forecasting accuracy between participants ranging from |3.28| to |40.56| (see Figure 1B).

As Figure 1B (right side) shows, dispositional eating happiness had a significant impact on the degree of the discrepancy, *F*(2,70) = 15.45, *p* < 0.001, and *η* ^2^ = 0.31. A significant discrepancy between forecasted and in-the-moment eating happiness emerged within each foodiness group, *p* ≤ 0.001 with effects ranging between *d* = 2.42 for the low, *d* = 2.20 for the middle, and *d* = 1.81 for the high foodiness group (see Table 2). Participants with a high (*M* = |10.72|, *SD* = 5.92) or medium tendency to foodiness (*M* = |14.50|, *SD* = 6.58) were less prone to a bias than participants with a low foodiness tendency (*M* = |22.30|, *SD* = 9.21), *p* < 0.001, and *p* = 0.001, respectively.

### Variability of Eating Happiness and Forecasting Accuracy

#### Analysis of Individual Eating Occasions Across the 14-Day Study Period

Besides variability in forecasting accuracy between participants (see Figure 1), in-the-moment eating happiness showed a considerable variability within and between participants across all 2,898 eating occasions, as indicated by the ICC of 0.47 (see Table 1). The ICC value indicates that the observed variance in in-the-moment eating happiness was almost equally due to differences in how happy people were with their eating (between-person variability) and to how much people varied in their experienced happiness from one eating occasion to the next (within-person variability). Across participants, number of eating occasion was not significantly related to variability, *r* = −0.10 and *p* = 0.395.

**Figure 2:**
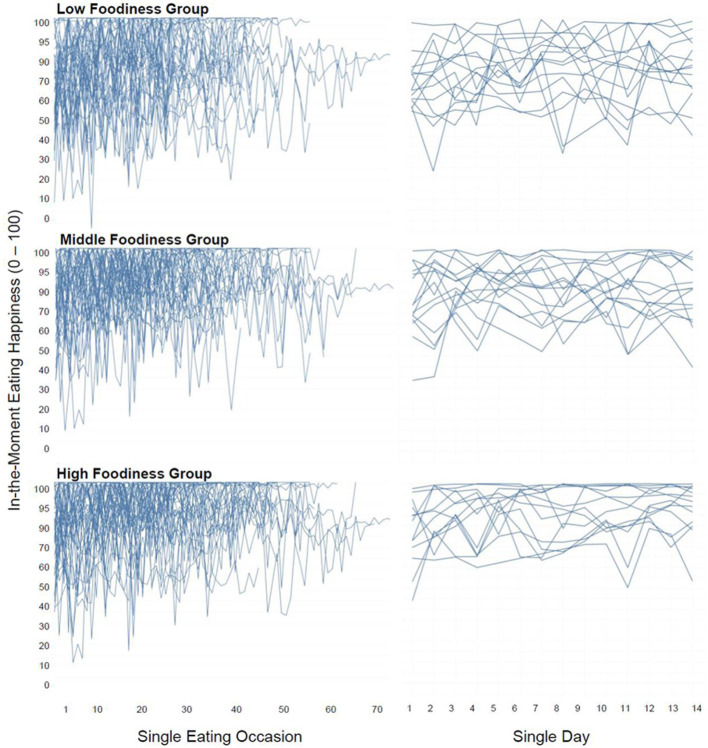
Variation of eating happiness experienced in-the-moment between eating occasions and between assessment days, separated by foodiness groups. Each participant is indicated by a separate line.

As the left side of Figure 2 shows, a substantial variation between and within individuals was also observed within each foodiness group (*ICC* = 0.40–0.42, Table 1). However, the three foodiness groups differed significantly in the variability of their in-the-moment eating happiness across eating occasions, *F*(2,70) = 4.57, *p* = 0.014, and *η* ^2^ = 0.12. People with a low tendency to foodiness showed greater variability of in-the-moment eating happiness (*M_Var_* = 217.04 and *SD_Var_* = 189.90), compared to people with a medium (*M_Var_* = 119.39, *SD_Var_* = 73.14, and *p* = 0.032) or high tendency to foodiness (*M_Var_* = 118.59, *SD_Var_* = 99.89, and *p* = 0.032).

Forecasting accuracy was significantly predicted by the variability of in-the-moment ratings, *β* = 0.30 and *p* = 0.010. The results indicate that a greater variability of in-the-moment eating happiness leads to a bigger discrepancy between forecasted and in-the-moment eating happiness. The variability of in-the-moment eating happiness explained 8.9% of the variance in forecasting accuracy (*F*(1,71) = 6.94, *p* = 0.010, and adjusted *R* ^2^ = 0.08). Even though foodiness groups differed significantly in their variability of in-the-moment eating happiness, the impact of variability on forecasting accuracy did not significantly differ across the three foodiness groups, *F*(2,5) = 1.44, *p* = 0.244, and adjusted *R* ^2^ = 0.30.

#### Analysis of the Day Level

As each participant ate several times a day, eating happiness was aggregated per day and variability between days was analyzed in a second step to consider possible compensatory effects between eating occasions within a day and secure a comprehensive analysis of the data. Similar to the variability observed between eating occasions, aggregated eating happiness per day also showed considerable variability between and within participants (see Figure 2, right side). The ICC value across participants (*ICC* = 0.67) indicates that 67% of the observed variance in in-the-moment eating happiness at the day level was due to differences between people, while 33% was due to how much eating happiness varied between days. However, the foodiness groups again differed significantly in the variability of their in-the-moment eating happiness across days, *F*(2,70) = 6.01, *p* = 0.004, and *η* ^2^ = 0.15. Variability on the day level was significantly higher for people in the low foodiness group (*M_Var_* = 111.97 and *SD_Var_* = 109.83), compared to people in the middle (*M_Var_* = 57.46, *SD_Var_* = 40.07, and *p* = 0.029) or high foodiness group (*M_Var_* = 44.63, *SD_Var_* = 43.11, and *p* = 0.005). Furthermore, as the right side of Figure 2 shows, a substantial inter- and intra-individual variation was also observed within each foodiness group, with ICC values ranging from 0.57 for the low foodiness group and, 0.63 for the middle foodiness group to 0.68 for the high foodiness group.

In line with the observed results at the eating occasion level, forecasting accuracy was also significantly predicted by variability between assessment days, *β* = 0.34 and *p* = 0.003. In total, 11.4% of the variance of forecasting accuracy was explained by variability between assessment days [*F*(1,71) = 9.13, *p* = 0.003, and adjusted *R* ^2^ = 0.10]. Furthermore, the impact of variability on forecasting accuracy did not significantly differ across the three foodiness groups, *F*(2,5) = 2.12, *p* = 0.128, and adjusted *R* ^2^ = 0.32.

## Discussion

The present study investigated forecasting accuracy for a familiar day-to-day experience, comparing forecasted eating happiness with eating happiness experienced in-the-moment using an event-based ecological momentary assessment. A significant difference between forecasted and in-the-moment eating happiness was observed. This shows that people’s forecasted emotional reactions for both distinct, outstanding events and familiar day-to-day experiences are inaccurate. Furthermore, the magnitude of the discrepancy was affected by both dispositional expectations (“foodiness”) and the variability of the in-the-moment experience, demonstrating that both stable inter-individual differences and experience-specific aspects influence forecasting accuracy.

Interestingly, while the relative difference score between forecasted and in-the-moment experience did not reveal an impact bias in the present study, the analysis of the absolute difference demonstrated a large effect for the divergence between forecasted and in-the-moment experience across participants (*d* = 1.81). The tendency to mispredict the intensity and/or duration of an emotional event has usually been described as an overestimation of the emotional impact, such as overestimating the pleasure of a vacation or the disappointment of a romantic breakup (see e.g., Gilbert et al., 1998; Wirtz et al., 2003). However, data from the present study revealed a substantial number of both overestimations and underestimations of in-the-moment eating happiness, explaining why the relative difference score did not reveal an impact bias across participants. One reason for this mixed forecasting pattern in this study might be the nature of the forecasted event. While distinct and outstanding events such as vacations or romantic breakups typically have uniformly positive or negative connotations across individuals, eating happiness is characterized by a greater inter- and intra-individual variance, meaning that eating experiences can vary both in their valence and in their intensity across as well as within individuals. General mechanisms such as focusing on central aspects (Wilson et al., 2000) or underestimating adaption over time (Gilbert et al., 1998) can provide an explanation for the absolute error, but the absolute error can be both to the positive and negative. The present study revealed an effect which is substantially higher than previously reported, for example, by Wirtz et al. (2003) with *d* > 0.61 from examining students’ real-life vacation experiences. However, the observed effect size is comparable to effect sizes in studies which also analyzed the absolute value of the discrepancy. For example, Hoerger et al. (2012a) found a significant discrepancy with an effect of *d* = 2.84 when comparing forecasted and in-the-moment experiences related to emotion-evoking pictures. This suggests that, examining an experience with no uniform connotation across individuals, the relative difference might reveal no impact bias across individuals not because people are able to provide accurate forecasts, but due to the prevalence of both overestimations and underestimations in forecasted reactions.

Further, day-to-day experiences are characterized by high familiarity and repetition, both possibly impacting the magnitude of the impact bias. The present data suggest that familiarity of the experience such as having previous experiences of an event or an emotional reaction does not necessarily improve forecasting accuracy. To learn from their emotional experiences, people must actively refer to and integrate relevant previous experiences into the process of forecasting (Wilson et al., 2001, 2003; Kermer et al., 2006; Ayton et al., 2007), which in turn necessitates an accurate recall of past emotional reactions. However, as the emotion itself is not stored in memory in a form that can be directly retrieved later (Robinson and Clore, 2002), past experiences are also subject to biases and people tend to overestimate their past emotional reactions (e.g., Redelmeier and Kahneman, 1996; Fredrickson, 2000). Furthermore, Robinson and Clore (2002) argue that the ability to learn from past experiences is impaired as details of our affective reactions become faded and less accessible over time, which in turn makes people rely more on general knowledge and beliefs when forecasting future affective reactions (see also Schwarz and Xu, 2011; Schwarz, 2012). In addition, the intensity of the impact bias might be so pronounced that it remains even after partial adjustment according to previous experiences, leading to biased forecasts (Wilson et al., 2001). The results of the present study, together with previous research, show that biases in forecasts are a general and robust phenomenon, prevalent for both outstanding and familiar events, with previous experience possibly moderating the magnitude of the bias, but not preventing it.

To further understand the impact bias, we analyzed the variability of the experience both between eating occasions within individuals and in relation to participants’ dispositional expectation toward eating (“foodiness”). One consequence of repeatedly eating throughout the day is a high number of distinct events that can vary both in valence and intensity. Forecasting an experience that involves a high fluctuation may be more difficult than a stable or consistent experience as people need to incorporate the variation of the experience across individual occasions. Focusing on the aspect of repetition within the experience shows that, as hypothesized, a greater variability of in-the-moment eating happiness resulted in lower forecasting accuracy across participants. Besides variations in the experience associated with food intake itself, people may also differ in their experience while eating, with some people enjoying and being happy with almost every food or meal and other people differentiating more between individual eating experiences.

To analyze this difference in experience while eating, we divided the sample into three groups based on the general expectation of eating (“foodiness”). The results showed that variability of in-the-moment eating happiness differed between foodiness groups with people in the low foodiness group displaying the greatest amount of variation between individual eating occasions. Expectations about an experience have been shown to affect the actual in-the-moment experience (Wilson et al., 1989; Klaaren et al., 1994; Totterdell et al., 1997; Wilson and Gilbert, 2003) and might therefore explain the difference in variability between foodiness groups. Specifically, differences in the variability might be explained by the affective expectation model (Wilson et al., 1989), according to which an affective reaction is formed by a comparison between expected and actual experience.

Geers and Lassiter (2002) further demonstrated that mental orientations toward experiences (optimism-pessimism) play an important role in the formation of in-the-moment experiences. People with a generally more positive outlook about their future (optimists) tend to assimilate their in-the-moment experiences toward their expectations, independent of whether their in-the-moment experience stands in line with or in contrast to their expectations. In contrast, people with a generally more negative outlook about their future (pessimists) have been shown to be more sensitive to contradicting information (Spirrison and Gordy, 1993). As a consequence, they only assimilate to their prior expectation when the experience is consistent with their expectation, while their affective reaction diverges from their expectation if they realize inconsistency (Wilson et al., 1989; Geers and Lassiter, 2002). Therefore, people with a low tendency toward foodiness might only have shown assimilation in congruent cases, while people with a high tendency toward foodiness might have assimilated toward their forecasted eating happiness regardless of whether or not their experience in-the-moment was consistent with their forecasts, leading to a more homogenous experience pattern and less variability.

However, even though variability of in-the-moment eating happiness differed between foodiness groups, the impact of variability on forecasting accuracy remained the same. Independent of dispositional expectations, experiencing more variability in-the-moment is more difficult to forecast, resulting in a lower forecasting accuracy. This indicates that forecasting accuracy is influenced by both stable differences between individuals, such as dispositions, but also by experience-specific differences such as the variability/stability of the experience. To summarize, dispositional expectation might influence the displayed variability of the in-the-moment experience, but the impact of the variability on forecasting accuracy is independent of dispositional expectations.

Findings of inter-individual differences also have implications on theories of affective forecasting. Most studies aim at examining and displaying errors at the general level across participants (see Wilson and Gilbert, 2003 for a review), focusing on mechanisms such as attention focus (Wilson et al., 2000) and rationalization processes (Gilbert et al., 1998). However, even though forecasts are prone to general mechanisms creating a systematic bias, a growing body of research provides evidence that people differ in their ability to provide accurate forecasts (Dunn et al., 2007; Wenze et al., 2012; Hoerger et al., 2012b; Christophe and Hansenne, 2016). Hoerger et al. (2016) suggest that dispositional differences such as personality contribute to forecasting accuracy because they affect underlying processes such as the ability to visualize the future, the awareness of the experience, and people’s tendency to forecast and experience more positive or negative emotions.

We add to this stream of research by assessing the impact of dispositional expectations as one facet of people’s personalities. The present results reveal that both in-the-moment experience and the magnitude of the bias are affected by people’s dispositional expectations toward eating (“foodiness”). Even though some people are better at forecasting their future affective responses, the pattern and variability of the actual experience play a crucial role in forecasting accurately. Consequently, summing up the independent effect for group membership and variability explains the greater divergence between forecasted and in-the-moment eating happiness in the low foodiness group. Hence, both dispositional differences and experience-specific aspects must be considered to enable meaningful conclusions for forecasting accuracy to be drawn.

From a broader perspective, it is interesting to relate the present findings to previous research on eating behaviors using ecological momentary assessment. Several studies focused on the relationship between affective reactions and eating behaviors (e.g., Liao et al., 2018; Strahler and Nater, 2018; Jeffers et al., 2019; Schultchen et al., 2019). However, their focus was primarily on the impact of stress and negative affect on food choice rather than the phenomenon of affective forecasting and how forecasted eating happiness relates to in-the-moment experienced happiness. However, integrating these lines of research appears promising and future research may specifically assess the degree to which variability of eating happiness can be attributed to situational context variables such as daily stress or emotional states (see Loewenstein, 1996; Loewenstein and Schkade, 1999; Gilbert et al., 2002; Loewenstein et al., 2003). In a related vein, the present study did not collect data on participant’s familiarity with the consumed foods. It seems possible that a diet composed of a rather limited and stable number of food items is easier to forecast than forecasting experiences with a greater variety and new and unknown foods and cuisines. Thus, future research should consider actual food intake to assess the effects of diet composition on the variability of in-the-moment eating happiness. It needs also to be considered that our results are based on a generally healthy sample that was interested in exploring their eating behavior. Furthermore, even though the sample size is comparable to or even larger than in other EMA studies assessing eating behaviors (Stein and Corte, 2003; Zepeda and Deal, 2008; Schüz et al., 2015a, 2015b), the sample might be considered as rather small in order to detect between-person effects (Gignac and Szodorai, 2016). Thus, the study findings should be replicated using larger and representative samples to acknowledge that eating is a complex behavior that is impacted by various factors and aspects on the personal, situational, and societal level (Renner et al., 2012; Stok et al., 2017). In addition, the finding that forecasted and actual experience often diverges may have potential implications for eating behaviors. For instance, while people adhere to the general belief that unhealthy foods lead to high pleasure (Raghunathan et al., 2006), in-the-moment eating happiness assessments revealed that fruits and vegetables evoked comparable high eating happiness as stereotypical unhealthy foods such as cake or candy (see Wahl et al., 2017a). Thus, one future direction of this line of research could build upon differences between forecasted and in-the-moment experiences to promote healthy eating.

In conclusion, the results of the present study contribute to the generalizability of research on affective forecasting, demonstrating that biased forecasts are a general phenomenon, present not only for outstanding events but also for familiar day-to-day experiences. Furthermore, dispositional differences between people such as dispositional expectations (“foodiness”) and experience-specific aspects such as variability/stability of the in-the-moment experience are both shown to be of great importance, with both impacting forecasting accuracy. Overall, while biased forecasts appear as a stable phenomenon in affective forecasts, inter-individual differences, and experience-specific aspects have a substantial impact in the manifestation and magnitude, and differentiated analyzes are therefore needed in research about affective forecasting.

## Data Availability Statement

The dataset generated during the current study and analyzed for the present article is available from the corresponding authors on reasonable request.

## Ethics Statement

The studies involving human participants were reviewed and approved by and pre-registered at the DRKS (ID DRKS00010279) and conformed to the guidelines of the German Psychological Society (Deutsche Gesellschaft für Psychologie) and the Declaration of Helsinki. The study protocol was approved by the University of Konstanz’s Institutional Review Board, and adhered to ethical guidelines and regulations. The participants provided their written informed consent to participate in this study.

## Author Contributions

BR and HS were responsible for study concept and design. BR, HS, KV, DW, LK, and KZ developed the concept for the mobile application “SMARTFOOD” and SB, JM, and HR were responsible for the HCI design and technical implementation. KV, DW, LK, and KZ were responsible for participant recruitment and data collection, under supervision of BR and HS. KV did the statistical analyzes with input from BR. KV and BR drafted the manuscript with important intellectual input from HS. All authors provided critical comments for and approved the final version of the manuscript for submission.

## Conflict of Interest

The authors declare that the research was conducted in the absence of any commercial or financial relationships that could be construed as a potential conflict of interest.

## Footnotes

^1^www.uni-konstanz.de/smartact
